# The Couple Dimension of Breast Cancer: Relationship Between Marital Functionality and Quality of Life in Women With Breast Cancer

**DOI:** 10.7759/cureus.106494

**Published:** 2026-04-05

**Authors:** Gloria Y Santos-Herrera, Nallely Rincón-Peregrino, Aziz Curioca-Velásquez, Alfredo González-Ayala, Hugo O Márquez-Villegas, Quitzia L Torres-Salazar

**Affiliations:** 1 Family Medicine, Hospital General de Zona No. 2 con Medicina Familiar del Instituto Mexicano del Seguro Social (IMSS), Salina Cruz, MEX; 2 Biomedical Sciences, Universidad Juárez del Estado de Durango, Durango, MEX

**Keywords:** breast cancer, couple relationship, marital functioning, oncology care, psychosocial factors, quality of life

## Abstract

Background

Breast cancer significantly affects the physical, emotional, and social well-being of affected women. Beyond clinical factors, interpersonal dynamics (particularly marital functioning) may influence how patients cope with the disease and perceive their overall quality of life. However, evidence regarding the relationship between marital functionality and quality of life in women with breast cancer remains limited in Mexican populations.

Objective

This study aims to evaluate the relationship between marital functionality and quality of life in women with breast cancer receiving follow-up care in an oncology outpatient clinic.

Methods

An observational, cross-sectional, and prospective study was conducted among women with a confirmed diagnosis of breast cancer attending the oncology outpatient clinic of the General Hospital of Zone with Family Medicine No. 2 of the Mexican Social Security Institute (IMSS) in Salina Cruz, Oaxaca, Mexico. Marital functionality was assessed using the Víctor Chávez scale, and quality of life was evaluated through a standardized quality-of-life instrument. Descriptive statistics were calculated using medians and interquartile ranges (q25-q75). Group comparisons were performed using nonparametric tests, and correlation between variables was analyzed using Spearman’s rho.

Results

A total of 110 women with breast cancer were included. Patients with poor quality of life showed a median marital functionality score of 45 points (q25-q75: 40-70), whereas those with good quality of life presented a significantly higher median score of 70 points (q25-q75: 50-95) (p = 0.002). When quality of life was analyzed according to the level of marital functionality, progressively lower scores were observed among women with moderate and severe marital dysfunction. Correlation analysis demonstrated a moderate positive association between marital functionality and quality of life (Spearman’s rho = 0.353, p < 0.001).

Conclusion

Marital functionality was significantly associated with quality of life among women with breast cancer. Patients with functional partner relationships reported better well-being, whereas marital dysfunction was linked to poorer outcomes. These findings highlight the importance of integrating psychosocial and couple-centered approaches into comprehensive oncologic care to improve the overall quality of life of women living with breast cancer.

## Introduction

Breast cancer currently represents one of the most significant public health challenges worldwide due to its high incidence, mortality, and social impact. It is recognized as the most frequently diagnosed malignant neoplasm among women and one of the leading causes of cancer-related death in the female population. Globally, approximately 1.7 million new cases are diagnosed each year, and the incidence continues to increase despite advances in early detection and oncologic therapies [1]. In Mexico, the epidemiological pattern mirrors global trends, with breast cancer representing the most prevalent malignant tumor among women and a major priority for health systems. These figures highlight the magnitude of the disease and underscore the need for comprehensive approaches that consider not only biomedical aspects but also the psychosocial determinants influencing patients’ experience of illness [2].

The clinical course of breast cancer frequently involves prolonged therapeutic trajectories that combine surgery, chemotherapy, radiotherapy, endocrine therapy, and targeted treatments. Although these interventions have improved survival, they are often associated with adverse effects that significantly affect physical and emotional well-being. Fatigue, pain, alterations in body image, fear of recurrence, and uncertainty regarding prognosis are common experiences reported by patients [3]. These factors contribute to psychological distress, including anxiety and depressive symptoms, which have been widely documented in the international literature as key determinants of health-related quality of life in women with breast cancer [4].

Beyond the individual experience of disease, breast cancer can also affect interpersonal relationships, particularly within the couple. The diagnosis and treatment of cancer may alter communication, intimacy, and emotional support, influencing the patient’s psychological adaptation and overall well-being. In addition, many patients experience cognitive changes associated with systemic therapy, commonly referred to as “chemo brain,” characterized by impairments in memory, attention, processing speed, and executive function. These alterations have been linked to neuroinflammatory processes, white matter changes, oxidative stress, and neurotransmitter dysregulation and may lead to frustration, anxiety, and reduced self-confidence, further affecting social interaction and couple dynamics [5].

The interaction between breast cancer, psychosocial factors, and quality of life is therefore best understood within a biopsychosocial framework, where biological processes, emotional responses, and social support systems (including the partner relationship) collectively shape the experience of illness. Conceptual models such as those proposed by Wilson and Cleary, as well as Ferrans and Powers, highlight the role of the social environment in modulating perceived health status and quality of life [6]. Despite increasing recognition of this relational dimension, the association between marital functionality and quality of life in women with breast cancer remains insufficiently explored in many clinical settings. Therefore, this study aimed to evaluate the association between marital functionality and quality of life in women with breast cancer receiving outpatient oncologic care.

## Materials and methods

Study design and setting

This study was conducted and reported in accordance with the Strengthening the Reporting of Observational Studies in Epidemiology (STROBE) guidelines [7]. An observational, cross-sectional study with prospective data collection was carried out to evaluate the relationship between marital functionality and quality of life in women with breast cancer. The study was conducted in the oncology outpatient clinic of the General Hospital of Zone with Family Medicine No. 2 of the Mexican Social Security Institute (IMSS) in Salina Cruz, Oaxaca, Mexico. The study population consisted of women with a confirmed diagnosis of breast cancer who attended follow-up consultations during the study period. Data were collected between June and December 2025.

Participants (patient recruitment)

Participants were recruited through consecutive sampling during routine outpatient consultations. Eligible participants were women aged 40-70 years with a confirmed diagnosis of breast cancer who were currently living with a partner. All eligible patients were invited to participate, and none declined participation during the study period. Participants were included after providing written informed consent. Women who were unable to complete the questionnaires due to cognitive impairment or severe clinical deterioration were excluded.

Data collection and instruments

Quality of life was assessed using the Functional Assessment of Cancer Therapy-Breast (FACT-B) questionnaire, a validated instrument widely used to evaluate health-related quality of life in women with breast cancer. The FACT-B consists of 37 items organized into five domains: physical well-being, social/family well-being, emotional well-being, functional well-being, and additional concerns related specifically to breast cancer. Each item is scored on a five-point Likert scale ranging from 0 (“not at all”) to 4 (“very much”), generating a total score that reflects the overall quality of life. The scoring procedure included inversion of negatively worded items according to the instrument guidelines. The FACT-B has demonstrated high internal consistency in similar populations, with Cronbach’s alpha coefficients exceeding 0.84, supporting its reliability and validity [8]. In the absence of standardized thresholds for the FACT-B, quality of life was operationalized as a dichotomous variable using a median split approach. Participants were classified as having poor or good quality of life according to whether their scores fell below or at/above the sample median, respectively.

Marital functionality was evaluated using the Chávez-Velazco scale, an instrument developed and validated in Mexico to assess the functioning of the marital subsystem [9,10]. This scale evaluates multiple dimensions of couple dynamics, including communication, affective expression, role distribution, sexual satisfaction, and shared decision-making, allowing a comprehensive assessment of relational functioning within the couple.

Data collection was conducted through individual administration of both questionnaires during routine consultations. The instruments were explained to each participant, who subsequently completed them independently in a private and comfortable setting. A member of the research team remained available to resolve any questions, ensuring adequate comprehension while minimizing potential bias. To ensure confidentiality, a unique identification code was assigned to each participant, and no personally identifiable information was recorded. All data were stored in a secure database with restricted access.

Sample size calculation

The sample size was calculated a priori using the formula for proportions in finite populations. A total population of 150 women with breast cancer under follow-up was considered. Assuming a confidence level of 95% (Z = 1.96), an expected prevalence of marital dysfunction of 60% based on previous studies, and a precision of 5%, the minimum required sample size was estimated at 107 participants. The final sample included 110 participants, exceeding the minimum required sample size.

Statistical analysis

All statistical analyses were performed using SPSS version 27 (IBM Corp., Armonk, NY). Quantitative variables were assessed for normality and described using appropriate measures of central tendency and dispersion, while categorical variables were summarized as frequencies and percentages. Differences between groups were analyzed using nonparametric tests as appropriate. The association between marital functionality and quality of life was assessed using Spearman’s rank correlation coefficient (Spearman’s rho), given the ordinal nature of the variables and the non-normal distribution of the data. Statistical significance was established at a p-value ≤ 0.05.

Ethical considerations

The study protocol was reviewed and approved by the Local Health Research Committee of the Mexican Social Security Institute (IMSS) under registration number R-2024-2001-044. All procedures were conducted in accordance with the ethical principles of the Declaration of Helsinki, and all participants provided informed consent before inclusion in the study.

## Results

A total of 110 women with breast cancer were included in the study. The mean age of the participants was 53.5 ± 11 years. Most patients were married (80%), while 20% reported cohabiting with their partner. The median duration of cohabitation was 27.5 years (q25-q75: 15-35), with a range of 5-51 years. The median time since breast cancer diagnosis was 30 months (q25-q75: 14-65.7), ranging from 1-330 months. Regarding clinical stage at diagnosis, most patients were classified as stage II (45.5%) or stage III (43.6%), whereas smaller proportions corresponded to stage I (4.5%) and stage IV (6.4%). At the time of evaluation, 59.1% of the patients were receiving active oncologic treatment, 30.0% were in remission, 9.1% had disease recurrence, and 0.9% were receiving palliative care (Table 1).

**Table 1:** Baseline characteristics of the study population (n = 110) *Continuous variables are presented as median (q25–q75), and **categorical variables are presented as frequencies and percentages. The majority of participants were married, with long-term relationships, and most were in intermediate clinical stages and receiving active treatment.

Variables	Value
Sociodemographic characteristics	
Age (years)*	53.5 ± 11
Marital status**	
Married	88 (80%)
Cohabiting	22 (20%)
Time living with partner (years)*	27.5 (15–35)
Disease-related characteristics	
Time since breast cancer diagnosis (months)*	30 (14–65.7)
Clinical stage of breast cancer**	
Stage I	4.5%
Stage II	45.5%
Stage III	43.6%
Stage IV	6.4%
Current clinical status	
Newly diagnosed (no treatment)	0.9%
Active treatment	59.1%
Remission	30.0%
Recurrence	9.1%
Palliative care	0.9%

The evaluation of marital functionality showed that only one-third of the participants (33.6%) presented a functional marital relationship. In contrast, 37.3% of the patients were classified as having moderate marital dysfunction and 29.1% as severe marital dysfunction, indicating that 66.4% of the women in the study experienced some degree of impairment in couple functioning (Table 2).

**Table 2:** Marital functionality distribution (n = 110) Data are presented as frequencies and percentages according to the Chávez-Velazco scale. Most participants showed some degree of marital dysfunction, with moderate and severe categories being the most frequent.

Marital functionality category	n	%
Functional	37	33.6
Moderately dysfunctional	41	37.3
Severely dysfunctional	32	29.1

**Figure 1:**
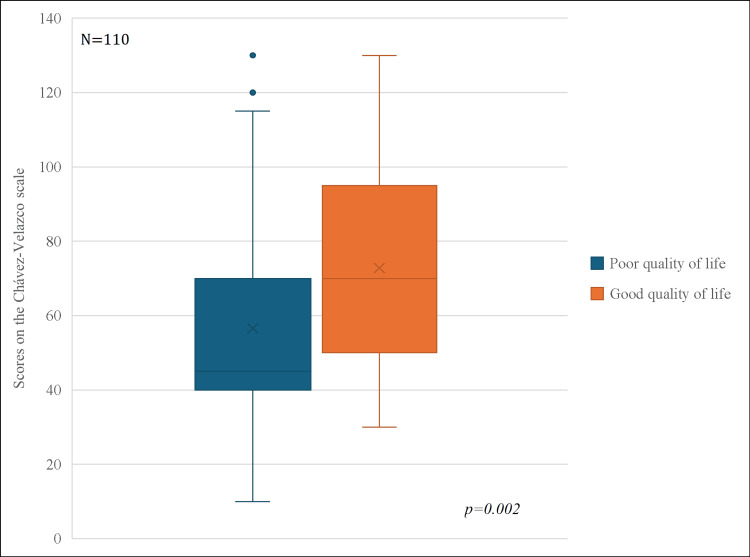
Score on the Víctor Chávez scale according to quality of life Box plot showing the distribution of Víctor Chávez scale scores according to quality of life (poor vs good). Boxes represent the interquartile range (q25–q75), the line indicates the median, and whiskers represent minimum and maximum values. Patients with good quality of life showed higher scores compared with those with poor quality of life (p = 0.002, Mann–Whitney U test).

When marital functionality scores were compared according to quality-of-life status, women with poor quality of life showed a median score of 45 points (q25-q75: 40-70) on the Víctor Chávez scale, whereas those with good quality of life presented a significantly higher median score of 70 points (q25-q75: 50-95). This difference was statistically significant (p = 0.002), indicating that a better quality of life is associated with higher levels of marital functionality (Figure 1).

**Figure 2:**
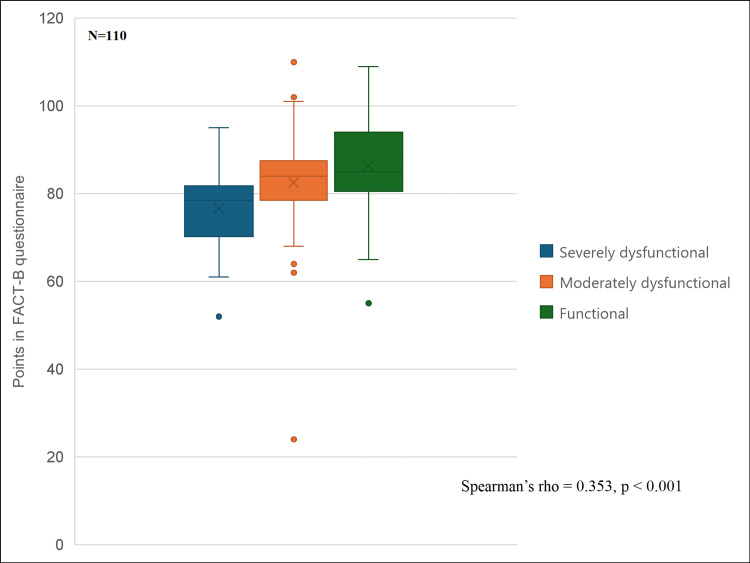
Quality-of-life scores according to level of family functioning Box plot showing quality-of-life scores across marital functionality categories (functional, moderately dysfunctional, and severely dysfunctional). Boxes represent the interquartile range (q25–q75), the line indicates the median, and whiskers represent minimum and maximum values. A progressive decrease in quality of life is observed as marital functionality decreases (Spearman’s rho = 0.353, p < 0.001). FACT-B: Functional Assessment of Cancer Therapy-Breast.

When quality-of-life scores were analyzed according to the level of marital functionality, a progressive decrease in quality of life was observed as marital functionality decreased. Patients with functional relationships showed the highest quality-of-life scores, whereas those with moderate and severe dysfunction presented progressively lower values. Overall, correlation analysis demonstrated a moderate positive association between marital functionality and quality of life (Spearman’s rho = 0.353, p < 0.001) (Figure 2).

## Discussion

In the present study, most women with breast cancer reported an overall quality of life within acceptable ranges, although physical and emotional domains were the most affected. Importantly, patients with functional marital relationships showed higher quality-of-life scores compared with those in moderately or severely dysfunctional relationships. These findings support the hypothesis that couple dynamics constitute a relevant determinant of well-being among women undergoing oncologic follow-up. Evidence increasingly suggests that health-related quality of life is influenced not only by clinical factors or treatment characteristics but also by the interpersonal and emotional context in which the disease is experienced.

In our sample, 59.1% of patients were receiving active oncologic treatment, while 30% were in remission, and only 0.9% were in palliative care. These findings are consistent with those reported by Mokhtari-Hessari et al., who have shown that quality of life in women with breast cancer varies according to the stage of disease and treatment phase, being generally lower during active treatment and improving during remission. This pattern is likely related to the cumulative adverse effects of systemic therapies, including fatigue, hematologic toxicity, gastrointestinal symptoms, neuropathies, and changes in body image, which may negatively affect both physical and emotional well-being [11].

Regarding marital functioning, 37.3% of couples were classified as moderately dysfunctional and 29.1% as severely dysfunctional. These findings are comparable to those reported by Soto-Araujo et al., who described a high prevalence of family dysfunction among women with breast cancer. The diagnosis of cancer often represents a critical life event capable of disrupting family and couple dynamics through role redistribution, financial stress, and emotional burden. Differences with other studies reporting higher proportions of functional families may be explained by contextual factors such as earlier disease stages or greater availability of psychosocial support services [12].

In terms of quality of life, 64.5% of patients reported good quality of life, whereas 35.5% reported poor quality of life. These findings are consistent with those described by Heidary et al., who highlighted the protective role of family support and partner relationships in maintaining positive perceptions of quality of life despite the physical and psychological burden of cancer. In this context, supportive couple relationships may facilitate emotional coping, promote treatment adherence, and encourage adaptive self-care behaviors [13].

Correlation analysis revealed a moderate relationship between marital functionality and quality of life. Similar findings have been reported by Hernández-Herrera et al., who observed that better marital functioning is associated with adaptive coping strategies such as emotional expression and social support. From a broader psychosocial perspective, dysfunctional relationship dynamics have been linked in previous literature to increased psychological stress, which has been associated with neuroendocrine and immune alterations, including dysregulation of the hypothalamic-pituitary-adrenal axis and sleep disturbances, potentially impacting both psychological and physical health [14].

International studies provide additional context for these findings. Garg et al. reported that more than 80% of premenopausal women experience persistent sexual dysfunction following breast cancer treatment, particularly after mastectomy or hormonal therapy. In our study, sexual satisfaction was also one of the most affected domains, suggesting that treatment-related hormonal changes and body image alterations have a consistent impact on intimate relationships across different populations [15].

Similarly, Jehan et al. observed that sexual and emotional consequences of breast cancer may be intensified in sociocultural contexts where communication about sexuality remains limited. This observation is particularly relevant for Latin American populations, where traditional gender norms may restrict open discussion of sexual concerns within the couple. Cultural factors may therefore modulate the way patients experience relational difficulties during cancer treatment [16]. In the same direction, Valente et al. reported that the relational experience of breast cancer is structured around key dyadic domains, including coping strategies, psychosocial support, communication, sexual life, and spirituality, all of which influence the couple’s psychological adjustment to the disease [17].

Intervention studies further support the importance of addressing couple dynamics in oncologic care. Cucciniello et al. reported that psychoeducational programs, cognitive-behavioral therapy, and couple-centered interventions significantly improve both marital adaptation and quality of life in breast cancer survivors. In contrast, these types of structured interventions remain limited in many health systems, which may contribute to the persistence of relational difficulties observed in clinical practice [18].

Likewise, Duan et al. reported that positive dyadic coping in couples with breast cancer positively predicts post-traumatic growth in both patients and their spouses, whereas negative dyadic coping exerts the opposite effect. These findings reinforce the clinical value of strengthening couple-based coping processes as part of comprehensive oncologic care [19].

Finally, analysis of the Víctor Chávez scale and quality-of-life domains indicated that sexual satisfaction and emotional well-being were the most affected areas. These findings are consistent with international evidence indicating that sexual dysfunction, body image disturbances, and emotional distress are among the most frequent long-term consequences of breast cancer treatment. Such changes have been associated with alterations in self-perception, intimacy, and communication within the couple, which may be related to lower quality-of-life scores.

Overall, our findings highlight the importance of considering the relational dimension of breast cancer. Although causal relationships cannot be established, these results support the integration of couple-focused interventions, sexual health counseling, and psychosocial support into comprehensive oncologic care as potentially beneficial strategies to address emotional well-being and relational functioning.

This study has some limitations that should be considered when interpreting the findings. Its cross-sectional design does not allow for causal inferences between marital functionality and quality of life; however, it provides a useful snapshot of their relationship in a real-world clinical setting. The study was conducted in a single center, which may limit external generalizability, although it reflects the characteristics of patients receiving care in a representative public healthcare institution. Additionally, the categorization of quality of life for analytical purposes may have reduced variability, although the primary analysis was based on correlation measures using continuous data. Furthermore, the use of self-reported instruments may be subject to response bias; however, this was minimized through private, self-administered questionnaires and anonymized data handling. Despite these considerations, the findings offer meaningful evidence on the role of marital functionality in the quality of life of women with breast cancer.

## Conclusions

Marital functionality showed a significant correlation with quality of life among women with breast cancer. Patients with functional relationships had higher quality-of-life scores, whereas those with moderate or severe marital dysfunction exhibited lower scores. These findings underscore the relevance of couple dynamics as an important psychosocial dimension of well-being in oncologic patients. Although the cross-sectional design precludes causal inference, the results support the consideration of couple-centered and psychosocial approaches as part of comprehensive breast cancer care.
